# Searching for evolutionary distant RNA homologs within genomic sequences using partition function posterior probabilities

**DOI:** 10.1186/1471-2105-9-61

**Published:** 2008-01-28

**Authors:** Usman Roshan, Satish Chikkagoudar, Dennis R Livesay

**Affiliations:** 1 Department of Computer Science, New Jersey Institute of Technology, Newark, NJ, USA; 2 Department of Computer Science and Bioinformatics Research Center, University of North Caroline at Charlotte, Charlotte, NC, USA

## Abstract

**Background:**

Identification of RNA homologs within genomic stretches is difficult when pairwise sequence identity is low or unalignable flanking residues are present. In both cases structure-sequence or profile/family-sequence alignment programs become difficult to apply because of unreliable RNA structures or family alignments. As such, local sequence-sequence alignment programs are frequently used instead. We have recently demonstrated that maximal expected accuracy alignments using partition function match probabilities (implemented in Probalign) are significantly better than contemporary methods on heterogeneous length protein sequence datasets, thus suggesting an affinity for local alignment.

**Results:**

We create a pairwise RNA-genome alignment benchmark from RFAM families with average pairwise sequence identity up to 60%. Each dataset contains a query RNA aligned to a target RNA (of the same family) embedded in a genomic sequence at least 5K nucleotides long. To simulate common conditions when exact ends of an ncRNA are unknown, each query RNA has 5' and 3' genomic flanks of size 50, 100, and 150 nucleotides. We subsequently compare the error of the Probalign program (adjusted for local alignment) to the commonly used local alignment programs HMMER, SSEARCH, and BLAST, and the popular ClustalW program with zero end-gap penalties. Parameters were optimized for each program on a small subset of the benchmark. Probalign has overall highest accuracies on the full benchmark. It leads by 10% accuracy over SSEARCH (the next best method) on 5 out of 22 families. On datasets restricted to maximum of 30% sequence identity, Probalign's overall median error is 71.2% vs. 83.4% for SSEARCH (P-value < 0.05). Furthermore, on these datasets Probalign leads SSEARCH by at least 10% on five families; SSEARCH leads Probalign by the same margin on two of the fourteen families. We also demonstrate that the Probalign mean posterior probability, compared to the normalized SSEARCH Z-score, is a better discriminator of alignment quality. All datasets and software are available online.

**Conclusion:**

We demonstrate, for the first time, that partition function match probabilities used for expected accuracy alignment, as done in Probalign, provide statistically significant improvement over current approaches for identifying distantly related RNA sequences in larger genomic segments.

## Background

The importance of RNA within cellular machinery and regulation is well established [1,2]. Consequently, a proper understanding of RNA structure and function is vital to a more complete understanding of cellular processes. It is conjectured that the human genome contains several thousand yet undiscovered ncRNAs that play critical roles throughout the cell. Profile-sequence and structure-sequence methods, such as HMMER [3] and INFERNAL [4], are commonly used to identify RNA homologs within much larger genomic segments. However, the requirement of a reliable family alignment and/or structure diminishes the utility of these approaches. This can happen especially when searching for evolutionary distant homologs or the query RNA sequence is surrounded by unalignable flanking nucleotides. In fact, homologous sequences below 60% pairwise identity are generally too difficult for current methods [5]. Simple pairwise alignment approaches are commonly used when sufficient familial data is not available. The SSEARCH program [6], a popular implementation of the Smith-Waterman algorithm, is frequently used for finding RNA homologs in genomic sequences. Moreover, it is a commonly used benchmark that new homology search methods are compared against [7-10]. The NCBI BLAST program [11], which is also a local alignment algorithm, is faster than SSEARCH but much less sensitive.

SSEARCH and BLAST both search for *optimal *local alignments, with BLAST sacrificing sensitivity for speed. Conversely, the maximal expected accuracy approach is based on *suboptimal *alignments. Here, sequences are aligned using posterior/match probabilities within pairwise alignments. These probabilities can be computed using partition function dynamic programming matrices, introduced by Miyazawa [12] and later studied by others [13,14], or pairwise HMMs as done in ProbconsRNA [15]. Partition function posterior probabilities are analogous to nucleotide-nucleotide frequency counts estimated from an ensemble of suboptimal alignments (see ref. [14] for more details). We recently implemented the partition function approach within the program Probalign [14], which outperforms other leading multiple aligners (Probcons [15], MAFFT [16], and MUSCLE [17,18]) on three different protein alignment benchmarks (BAliBASE [19], HOMSTRAD [20], and OXBENCH [21]).

While Probalign was designed for global alignment, its performance on datasets of heterogeneous length [14] suggests an affinity for local alignment. Here, we implement a slightly modified Probalign version attuned to local alignment search. We have studied its performance on the pairwise RNA-genome homology search problem for divergent sequences and when the query is flanked by genomic nucleotides. We compare it to SSEARCH, BLAST, ClustalW [22], and HMMER (with single sequence profiles). We include ClustalW (with zero end gaps) due its wide usage in solving different alignment problems. In addition, ClustalW serves as an analogous example of a global multiple alignment method applied to this problem for us to compare Probalign to. We have carefully constructed a benchmark of divergent RNA-genomic alignments using real DNA and RNA sequences from the EMBL [23] and RFAM [24] databases, respectively. In order to maintain a reasonable level of difficulty and tractability for the experiments, each genomic sequence in our benchmark is at least 5K and at most 16K nucleotides in length. For added difficulty and to simulate practical conditions where exact 5' and 3' ends of ncRNA are unknown, we add real genomic flanks of size 50, 100, and 150 nucleotides to the query RNA of each dataset.

We specifically omit INFERNAL from this investigation for several reasons. First, and most importantly, (as discussed above) the utility of profile-sequence and structure-sequence alignment methods is limited by experimental data. At large evolutionary distances and with unalignable genomic flanks surrounding the query, which is the particular focus of this study, obtaining reliable RNA family alignments is considerably difficult. Second, the cmsearch program of the INFERNAL suite is, in part, used for constructing RFAM families from which we construct our benchmark. Additional sequences found using INFERNAL were added to the RFAM seed alignments [25]. Finally, we use cmsearch in intermediary steps of producing the benchmark (explained in the Methods Section below). In light of all these facts, it would be inappropriate to include INFERNAL in our experiments. We include HMMER in our experiments using both global-local and local-local alignment models (i.e. -g and -f, -s options); however, we construct the HMMER model using single sequence queries (without flanks) from the benchmark. In this way we have a reasonable comparison to the other sequence-based programs in our test set. In the remainder of the paper we refer to this setting of the program as just HMMER.

We find Probalign to have overall highest accuracies on the full benchmark. It leads by 10% accuracy over SSEARCH (the next best method) on 5 out of 22 families. On datasets restricted to maximum of 30% sequence identity, Probalign's overall median error is 71.2% vs. 83.4% for SSEARCH (the next best method). This difference has Friedman rank test P-value less than 0.05. Furthermore, on these datasets, Probalign leads SSEARCH by at least 10% on five families whereas SSEARCH leads Probalign by the same margin on two families out of a total of fourteen. We also demonstrate that the Probalign mean posterior probability, compared to the normalized SSEARCH Z-score, is a better discriminator of alignment quality. The Probalign mean posterior probability has Receiver Operator Characteristic (ROC) area under curve of 0.834 compared to 0.806 of the normalized SSEARCH Z-score.

Note that the performance of RNA homology search programs was examined previously by Freyhult et. al. [7]. Their benchmark and goals, however, were considerably different than ours. They studied RNA homology searches within RFAM RNA sequence databases without genomic flanks, and considered only a single genomic search example. Here, we are specifically interested in performance of programs for finding low sequence similarity RNA homologs (with flanks) in long genomic sequences.

## Results

We computed the mean error of each method within each RNA family by averaging over all pairwise alignment scores belonging to that family. We then computed the overall error of each method as the average score across all families.

### Full benchmark with query flanks

The full benchmark containing query RNAs with flanks constitute 13,716 datasets. We exclude HMMER when unalignable flanks are present since these will only confound the model. Table 1 lists the overall mean and median error of all methods on the full benchmark. Probalign's improvement is statistically significant lowest on datasets restricted to max 30% sequence identity. On these datasets it leads SSEARCH (the next best method) by 6.5% in mean error and 11.2% in median error.

**Table 1:** Mean and median percent error for all methods on the full benchmark (13,716 datasets) including query RNAs with flanks of size 50, 100, and 150.

Mean and median error	Probalign	SSEARCH	BLAST	ClustalW
Complete benchmark	**35.3 **| **30.7**	38.7 | 33.2	41.0 | 34.0	47.6 | 50.3
Datasets with pairwise sequence identity at most 30%	66.5* | 71.2*	73.0 | 83.4	75.9 | 85.3	82.9 | 85.0

BLAST does not return an alignment in 425 datasets and hence they are omitted from the calculations. HMMER is not shown since queries with unalignable flanks cannot be used to produce a reliable model. There are 14 families that contain datasets with at most 30% sequence identity. Probalign has overall lowest mean and median error. Bold indicates the best performance; the difference is larger on datasets with low sequence identity and significant with P-value < 0.05 (indicated by *).

Table 2 lists the error rates of Probalign and the next best method, SSEARCH, on each RFAM family. Probalign leads by 10% on a total of five families, namely T-box, Intron group I, signal recognition particle (eukaryotic), transfer RNA, and elenocysteine insertion sequence. The maximum improvement by SSEARCH over Probalign is on the U4 spliceosomal RNA family by 3.1%.

**Table 2:** Mean Probalign and SSEARCH percent error shown for each RFAM family in the full benchmark and for datasets with maximum pairwise sequence identity of 30%.

RFAM Family	Complete benchmark dataset			Subset with pairwise identity up to 30%		
	Probalign	SSEARCH	Difference	Probalign	SSEARCH	Difference

5S_rRNA	22.7	20.7	-2.0	*Zero datasets*		
U1 (4)	15.0	15.6	0.6	87.3	100.0	12.7
tRNA (256)	62.0	74.4	12.3	69.8	84.8	15.0
RNaseP_bact_a	34.0	33.0	-1.0	*Zero datasets*		
RNaseP_bact_b	29.0	29.1	-0.1			
U3	41.3	38.8	-2.5			
U4 (8)	25.3	22.2	-3.1	52.8	11	-41.8
SRP_euk_arch (132)	43.8	56.4	12.6	62.1	78.0	15.9
tmRNA (180)	32.0	36.3	4.3	50.5	59.8	9.4
Intron_gpI (4)	67.4	80.1	12.7	100.0	100.0	0.0
SECIS (208)	82.3	93.9	11.5	87.9	100.0	12.1
IRE (216)	44.4	48.7	4.2	88.7	96.5	7.7
THI	29.5	30.1	0.6	*Zero datasets*		
Hammerhead_1	43.7	46.0	2.3			
Purine (4)	16.2	16.4	0.2	17.4	1.8	-15.6
Lysine (16)	48.0	57.3	9.3	73.1	100.0	26.9
SRP_bact (80)	28.5	25.7	-2.8	62.6	65.0	2.3
SSU_rRNA_5 (4)	30.5	32.4	1.9	39	61	22
T-box	27.4	46.0	18.6	*Zero datasets*		
glmS (4)	23.4	21.0	-2.4	73.8	78.4	4.6
RNaseP_arch (8)	32.4	34.0	1.6	87	100.0	13
IRES_Cripavirus	5.7	3.9	-1.8	*Zero datasets*		

Unlike Table 1 above, where some datasets are omitted due to BLAST, all datasets of the benchmark are considered here. Difference is always calculated as the SSEARCH error minus Probalign error, meaning positive numbers indicates Probalign outperforms SSEARCH. Shown in parenthesis is the number of datasets in each family with maximum pairwise sequence identity of 30% (the same query RNA but with different flank sizes is considered a separate dataset).

Column two of the table lists the Probalign and SSEARCH error on datasets restricted to maximum 30% sequence identity. There are fourteen families containing datasets that satisfy this criterion. Out of the total fourteen, Probalign leads by at least 10% on five families whereas SSEARCH leads Probalign by at least same margin on two families.

In Table 3 we look at the effect of increasing query flank size on the accuracy of all methods. As expected, all methods yield higher error as the query RNA flank size increases. However, Probalign still has the statistically significantly lowest error (P-value < 0.05).

**Table 3:** Mean percent error as a function of query RNA flank size.

Query RNA flank size	Probalign	SSEARCH	BLAST	ClustalW
50	35.4*	39.3	41.9	48.5
100	36.8*	40.8	44.5	51.4
150	38.5*	43.3	45.9	53.2

For each flank size there are 3,429 datasets (see Methods Section for description of benchmark). As in Table 1 about 105 datasets per flank size are omitted on which BLAST does not return any output. Bold indicates the best performance and * indicates Friedman rank test P-value < 0.05.

### Benchmark without query flanks

In order to compare the programs against HMMER, we separate from the benchmark those datasets with no query RNA flanks (a total of 3,429). Each of these query RNAs can be used to specify a model in HMMER since misleading flanks are now absent. From Table 4 we see that HMMER does not perform very well with single sequence profiles, which is not surprising as using in this way (single sequence vs. multiple sequence profiles) clearly goes against its intended usage. On datasets restricted to maximum pairwise identity of 30% Probalign has the lowest mean and median error, leading by at least 18% over SSEARCH, the next best method.

**Table 4:** Mean and median percent error for all methods on the benchmark without query RNA flanks (3,429 datasets).

Mean and median error	Probalign	SSEARCH	BLAST	ClustalW	HMMER
Complete benchmark	**30.8 **| 30.4	31.4 | 22.1	32.0 | **20.9**	37.9 | 38.5	44.9 | 44.7
Datasets with pairwise sequence identity at most 30% (14)	**62.4 **| **59.5**	70.8 | 94.5	78.4 | 100.0	74.5 | 97.5	96.7 | 100.0

Probalign has lowest mean and median error on low sequence identity datasets.

### Discriminating true from false alignments

In order for the evaluated methods to be of practical utility in ncRNA searches, alignments found when there is no target-query match (a common real-world scenario), should be of poorer quality than the alignments above where target-query matches were always present. To assess the discriminative ability of Probalign and SSEARCH (the two best scoring methods above), we generated a false dataset of query-target pairs where the query and target were randomly selected from *distinct *RFAM families (see Sub-Section Alignment quality measures under the Methods Section). The size of the false dataset is 13,716, exactly the same as the real dataset used above. Concatenating the real and false datasets results in 27,432 target-query pairs that were subsequently aligned using both methods. An alignment on a false positive dataset or an alignment with 100% error on the benchmark is classified as a false positive. An alignment on the benchmark with less than 100% error is classified as a true positive. A good discriminator would have a high value on alignments with high accuracy and low value on alignments with 100% error on benchmark datasets or on the false positive dataset. In this case we are interested in the quality of the Probalign mean column posterior probability and the SSEARCH normalized Z-score as alignment discriminators.

**Figure 1:**
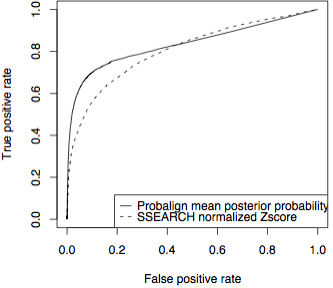
ROC curves for Probalign mean posterior probability and SSEARCH normalized Z-score. To construct this curve we added to our dataset a set of false hits by replacing each genomic sequence in each dataset of the benchmark with a randomly selected one from a benchmark dataset of a different RNA family. The ROC analysis clearly demonstrates that the Probalign is better able to discriminate true from false alignments.

In order to evaluate a discriminator, we need to set an *ad hoc *threshold. For example, we may choose to classify all alignments above 0.5 Probalign mean column posterior probability to be correct hits and incorrect otherwise. In order to eliminate the arbitrariness of such a definition, we employ Receiver Operating Characteristic (ROC) analysis. Along the ROC curve, true and false positive prediction values are plotted for a series of less stringent thresholds. The further the ROC curve is to the left, the better the method is; the diagonal indicates a method based on random guesses. As can be clearly seen in Figure 1, both methods perform significantly better than random. However, the analysis also clearly indicates that Probalign is better able to discriminate true from false target-query pairs. Probalign has an area under curve of 0.834 whereas SSEARCH has 0.806. The improved performance of Probalign is most striking at false positive rates between 2 and 40%.

### Computational running time and memory requirements

The current Probalign implementation is not as sophisticated as its SSEARCH counterpart, and therefore is much slower in comparison to the SSEARCH time. However, in practice it never takes more than a few seconds on any of our datasets. The average Probalign running time on the benchmark is 5.4 seconds compared to 0.04 seconds of SSEARCH, 0.5 seconds of ClustalW, 0.003 seconds of BLAST, and 0.14 seconds of HMMER (hmmsearch). These running times were computed on 2.4 GHz AMD Opteron 64 bit machines.

## Discussion

A standard technique for discovering new RNAs, in the absence of queries, is to align genomic fragments and search the alignment for significant structural conservation. QRNA [26] RNAz [27] and MSARI [28] are some well-known programs frequently used for this purpose. Their performance, of course depends upon the underlying sequence alignments. Our work here suggests that Probalign genomic alignments may align hidden (but related) RNA better than standard methods when given two genomic sequences. As a result it could produce more informative alignments for RNA detection programs such as the ones listed above.

Several improvements are currently underway to Probalign. A full Probalign-local implementation would include a Smith-Waterman implemenation of posterior probability local alignment, as done in the Proda [29] program. We expect such an implementation to produce better mean posterior probabilities estimates of the alignment quality since it would exclude unrelated genomic flanks.

In big-O notation Probalign's worst-case running time and memory requirements for pairwise alignment is O(mn) where m and n are the lengths of the input sequences. Probalign's memory requirements can be improved to O(mn^1/2^) with a 1.5 factor slowdown using memory reduction techniques used for HMM-based alignment programs [30]. This is part of planned future work.

Finally, it remains to be seen if Probalign partition function posterior probabilities demonstrate the same level of improvement seen her for the profile-sequence alignment and profile-profile alignment problems. The utility of profiles, however, is limited when unknown and unalignable genomic flanks are present or the family alignment is not rich or accurate enough. In that case, our current Probalign implementation offers a viable solution as demonstrated.

## Conclusion

This report represents the first examination of the Probalign alignment algorithm to search for RNA homologs within much larger genomic segments using partition function posterior probabilities. We show that the method does much better than the widely used SSEARCH and BLAST programs. Furthermore, the Probalign mean posterior probability (which has previously been discussed as a possible metric to assess alignment quality, but never studied carefully) has been shown to be a better indicator of alignment quality than the standard SSEARCH Z-score.

## Methods

### Benchmark

We began by extracting all 26 RFAM [24] seed alignments with known published RNA secondary structures and average pairwise sequence identity of at most 60%. During the benchmark construction process four families fail to meet length and uniqueness criteria (see below); this subsequently leaves us with 22 families in the end. At the time of writing of this paper RFAM version 7.0 was the most recent release. We measure sequence identity in regions of known secondary structure only, which are generally more reliably aligned than the rest. The 60% threshold has previously been identified as a cutoff for hard RNA alignment cases [5] and so we focus specifically on this region. We use the following three main steps to construct our benchmark from the initial 26 families.

#### 1. Pairwise RNA-RNA alignments

For each of the initial 26 RFAM seed multiple alignments, we randomly select a maximum of 350 pairwise alignments. In families where there are less than 350 total pairs, we consider them all.

#### 2. Construction of genomic flanks

Every RNA sequence in RFAM seed is cross-linked to a genomic sequence in EMBL [20]. For each pairwise alignment produced above, we randomly select one of the RNA sequences and attach real genomic flanks from EMBL (version r88) to each end of the RNA. Each genomic flank is truncated to 7500 base pairs on either end. Since the largest RNA sequence is at most 1000 nucleotides long, the maximum size of each genomic sequence is 16,000. This gives us RNA genome alignments where the RNA sequence can be considered as a *query *and the aligned homologous RNA is the *target *"hidden" in the genome. In order to make our dataset challenging enough, we exclude datasets where the genomic sequence is shorter than 5000 nucleotides.

#### 3. Alignment uniqueness

The attached genomic flanks may contain additional related RNAs of the same family as the query and the target (to which the flanks were attached). This means that two different correct alignments are possible. To keep things simple, we exclude such datasets and ensure that each query-target alignment is unique. For each dataset we built a profile from the RFAM family alignment annotated with consensus secondary structure using the cmbuild program of the INFERNAL suite. We then ran the cmsearch program of the INFERNAL suite on the genomic sequence of the dataset and excluded it entirely from the benchmark if more than one hit above a bit score of 30 was reported.

The pruning process yielded a total of 3,429 pairwise alignments distributed (unequally) among 22 RNA families. As mentioned earlier, all the datasets in four families failed to meet our length and uniqueness criteria just described. This subsequently leaves us with 22 families in the end. The 22 families and their characteristics can be found in Additional file 1.

#### Adding genomic flanks to query RNA

To simulate practical conditions where the exact 5' and 3' ends of ncRNAs are unknown, we took each dataset in our benchmark and produced three similar versions. However, in each of the three versions we added real 5' and 3' genomic flanks of size 50, 100, and 150 nucleotides to the query RNA of each dataset. By cross-referencing each RNA sequence to the original genomic version in EMBL we were able to obtain proper real genomic flanks and hence did not need to simulate artificial ones. Subsequently, the size of our benchmark increased four-fold from 3,429 to 13,716. We remove gaps from each alignment and use the flanked query and target genomic sequences as input to each program.

The full benchmark is available online [31]. Also available at the website are the RFAM family alignments from which the benchmark was created, training datasets (see below), and false positive datasets used for discrimination tests (described below).

### Alignment programs and parameters

#### Training data

We used a subset of the benchmark with query RNA flanks of size 100 nucleotides for training the program parameters. For each of the 22 divergent families we selected 25 random datasets. If the family contained a total of less than 25 pairwise alignments we included all in the training set. The final training set contained 498 pairwise alignments and can be found on the website for this paper [31].

#### Probalign

We used a modified version of the Probalign beta 1.0 program more attuned to local alignment. We make two modifications to the partition function matrices. They follow from analogous standard dynamic programming recursions for local alignment and can also be found in Muckstein et. al. [13]. First, we add 1 in the calculation of the *match *partition function matrix: $Z_{i,j}^{M}=(1+Z_{i-1,j-1}^{M}+Z_{i-1,j-1}^{E}+Z_{i-1,j-1}^{E})e^{s(x_{i},y_{j})/T}$. Second, we set the total partition function value to $Z=1+\sum_{i,j}Z_{i,j}^{M}$. The initial values of the *Z*-matrices also need to be set appropriately in line with the two changes. However, since we use zero end-gaps, this is automatically taken care of. We direct the reader to Additional file 1 for a more detailed description of the partition function matrices and notation. Probalign returns one alignment of the complete query against the genomic sequence. However, to produce multiple alignments of significant mean posterior probability, we provide a Perl script [31]. This script produces multiple alignments of the query against the genomic sequence by removing the aligned portion of the genome to the query and realigning the remainder to the query until the mean posterior probability is zero. In other words, all hits above zero probability are reported. This parameter can easily be modified in the script. We evaluate only the top hit in our experiments. We use the SSEARCH +5/-4 scoring matrix for Probalign and optimize gap open, gap extension penalties and the thermodynamic temperature on the training benchmark. The modified Probalign program is available as standalone code [31].

#### BLAST

We use the bl2seq program (current version 2.2.16) of the NCBI BLAST suite in our experiments. In our terminology we use BLAST to represent the bl2seq program of the suite. BLAST returns local alignments that may not include the entire query. In order to measure the error correctly, we require the entire query aligned to the genomic sequence (see Prediction Error Subsection below). We accomplish this by extending the local alignment in either direction until the full query is aligned to the genomic sequence. We also evaluate only the highest E-value BLAST hit. We actually tested the performance of the second hit outputted on each dataset and found that it had much worse error than the first. This is expected since each pairwise alignment in our benchmark is unique. We optimize BLAST gap parameters using both its default scoring matrix (+3/-1) and +5/-4 (the same one as used in SSEARCH). In order to avoid excessive scenarios where BLAST does not return an alignment, we set the minimum word size to 4. We use the +5/-4 matrix for BLAST since it performs better than the default (both with optimized parameters) on our training benchmark.

#### SSEARCH

We use the current SSEARCH release version 3.4t26 in our experiments. SSEARCH is a local alignment program and may not contain the entire query aligned to the genome (necessary for correct error computation). This problem can be fixed using the same BLAST treatment described above. With the -a option, however, SSEARCH returns alignments of both query and genome sequence in their entirety. In this case we find the accuracies to match those calculated otherwise, which is by fixing the alignments if necessary. Thus, without loss of any accuracy we run SSEARCH with -a enabled. We optimize the SSEARCH gap open and gap extension penalty parameters on the training benchmark. Like BLAST, we also found the second SSEARCH hit to be significantly much worse off than the first one.

#### ClustalW

We use ClustalW version 1.83 for our experiments. ClustalW, like Probalign, returns one global alignment of the complete query against the genomic sequence. We set the terminal gap (end-gap) penalties to zero. We optimize ClustalW gap parameters on the default ClustalW scoring matrix of +10/-9 and the SSEARCH +5/-4. However, the ClustalW default matrix optimal gap parameters perform better than the optimized +5/-4 matrix.

#### HMMER

We use HMMER version 2.3.2 in our experiments, the most current at the time of writing this paper. HMMER is designed for profile-based search that requires family alignments. Since we are interested in studying query RNA genomic search, particularly for divergent and hard cases where family alignments are not reliable, we use the single sequence RNA query for constructing the HMMER model. We use the hmmbuild program of the HMMER suite to build local alignment models (with the -f and -s options) and global alignment models (with the -g option). We then use the hmmsearch program on the training benchmark and each of the three models to search the genomic sequence for homologs of the query RNA. We find the local alignment -f and -s models to be equally the best performing and use -f in our experiments. Like BLAST and SSEARCH, HMMER local alignments may not contain the full query aligned to the genome. Therefore, we fix it in the same manner described above in the BLAST option.

Table 5 provides all parameters used in the four non-model based methods. The HMMER parameters are estimated from the single sequence profile specific to each dataset and therefore not included in Table 5. In Additional file 1 we list the exact command line options used for running our programs.

**Table 5:** Description of optimized parameters derived for each method used herein.

Method	Scoring matrix	Gap opening penalty	Gap extension penalty
Probalign	+5/-4 (T = 7)	32	2
SSEARCH	+5/-4	10	4
ClustalW	+10/-9	13	6
BLAST	+5/-4	8	6

### Alignment quality measures

#### Probalign mean posterior probability

The Probalign mean posterior probability is defined by Equation 1. *P(x*_*i *_~ *y*_*j*_*) *is the posterior probability of the *i*^*th *^nucleotide of sequence *x *aligning to the *j*^*th *^nucleotide of sequence *y*. More details about how this is computed and the Probalign method in general can be found in Additional file 1.

(1) $$\text{Probalign mean posterior probability }=\frac{\sum_{x_{i}\neq -,y_{j}\neq -}P(x_{i}\sim y_{j})}{(\text{\#}\text{​}\text{aligned nucleotides with non-zero posterior probability)}}$$

#### SSEARCH normalized Z-score

The SSEARCH Z-score and E-value are standard statistical measures of alignment reliability [32,33]. The Z-score can be compared across different sequence pairs [34]. We use the normalized Z-score as predictor of alignment quality. The normalized Z-score is the standard Z-score divided by the number of aligned nucleotides in the local alignment. We find this to produce a much better ROC analysis than the raw Z-score and the normalized and raw E-value.

#### False positive datasets

In order to measure the prediction accuracy of the above two measures we created a set of false positives. For each dataset in our benchmark, we create a false positive one by replacing the genomic sequence with one selected from a different random dataset. Now, each false positive dataset contains a query RNA and a genomic sequence containing a target RNA from a different family. We expect any alignment reliability measure to have a low value on these datasets. We make these datasets available online [31].

### Measure of accuracy and statistical significance

#### Prediction error

**Figure 2:**
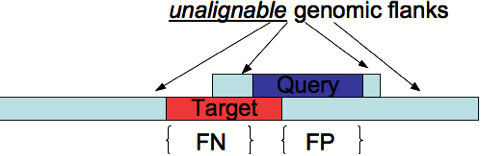
A cartoon of false positive and false negative situations for a query-target alignment.

We are interested in finding out how much of the target RNA (which lies in the genomic sequence) is aligned to the query, excluding the query flanks. As described above, for BLAST, SSEARCH, and HMMER, all of which return local alignments, we extend the query-genome alignment in both directions until the entire query, but not its flanks, is matched to the genomic sequence. This improves sequence coverage, reduces the false negative rate, and also allows a fair comparison to Probalign and ClustalW, both of which return global alignments of the entire sequences. For each method, we take the part of the genomic sequence aligned to the query in its alignment, and measure the false positive as the number of nucleotides in this region that are not in the target RNA. Similarly, we measure the false negatives as the number of nucleotides in the target RNA that are not in the genomic region aligned to the query (in the method estimated alignment). See Figure 2 for a visual description of the false positives and false negatives. We normalize the false positive and false negatives by the size of the genomic region aligned to the query in the computed alignment and the size of the target RNA respectively. The normalized false positive and false negatives can now be expressed as a percentage between 0 and 100. We measure the error, also expressed as a percentage, as the average of the normalized false positive and false negative.

#### Statistical significance

Statistically significant performance differences between the various alignment methods are calculated using the Friedman rank test [35]. This is a standard measure used for discriminating alignments in benchmarking studies [18,36]. Roughly speaking, lower P-values coincide with reduced likelihoods that the ranking differences are due to chance. We consider P-values below 0.05 (a standard cutoff in statistics) to be statistically significant.

#### Correlation with true hits and true accuracy

We conduct an ROC analysis [37] to study how well the Probalign mean posterior probability and the SSEARCH Z-score can predict the quality of the alignment. An ROC curve plots the true positive rate (y-axis) against the false positive rate (x-axis). The area under the curve is an indicator of overall accuracy of the classifier. All ROC area under curve values are normalized to 1 with higher areas indicating higher accuracy. We treat the Probalign mean posterior probability and the SSEARCH normalized Z-scores as classifiers for a true or false hit.

## Authors' contributions

Roshan conceived of the study and developed the benchmark. Livesay and Chikkagoudar contributed to the execution of the study and writing of the paper. All authors have read and approved the final version of the manuscript.

## Supplementary Material

Additional file 1 Supplementary material. Characteristics of RNA families used in this study, detailed description of Probalign, and command line parameters of programs used. Click here for file
