# Supplementary material for: Searching for evolutionary distant RNA homologs within genomic sequences using partition function posterior probabilities
Source: BMC Bioinformatics. 2008 Jan 28;9:61. doi: 10.1186/1471-2105-9-61 (PMC2248559; doi:10.1186/1471-2105-9-61)
Supplement: Additional file 1 — Supplementary material. Characteristics of RNA families used in this study, detailed description of Probalign, and command line parameters of programs used. [file 1471-2105-9-61-S1.DOC]

# Supplementary material

RNA-genome benchmark

Table 1 below lists some characteristics about the 22 RNA families in our benchmark. We first created subsets of each RFAM seed family alignment containing a maximum of 50 randomly selected sequences. For each subset we then follow the directions listed in the main paper to construct the benchmark.

**Table 1**: Statistics for all 22 RFAM RNA families used in our study

| RFAM RNA family | Average pairwise sequence identity | Sequence length standard deviation | Number of sequences in seed family alignment | Number of pairwise alignments in benchmark |
| --- | --- | --- | --- | --- |
| 5S_rRNA | 55 | 2.58 | 50 | 49 |
| U1 | 56 | 6.67 | 50 | 141 |
| tRNA | 39 | 4.9 | 50 | 342 |
| RNaseP_bact_a | 59 | 37.78 | 50 | 143 |
| RNaseP_bact_b | 59 | 37.84 | 50 | 23 |
| U3 | 45 | 55.94 | 21 | 20 |
| U4 | 56 | 11.04 | 26 | 69 |
| SRP_euk_arch | 45 | 10.45 | 50 | 331 |
| tmRNA | 40 | 31.51 | 50 | 342 |
| Intron_gpI | 43 | 77.46 | 30 | 71 |
| SECIS | 41 | 3.16 | 50 | 347 |
| IRE | 54 | 1.43 | 39 | 231 |
| THI | 55 | 17.99 | 50 | 347 |
| Hammerhead_1 | 56 | 31.95 | 50 | 49 |
| Purine | 50 | 0.85 | 12 | 59 |
| Lysine | 45 | 8.47 | 19 | 147 |
| SRP_bact | 50 | 9.19 | 42 | 348 |
| SSU_rRNA_5 | 48 | 128.30 | 50 | 97 |
| T-box | 51 | 2.49 | 14 | 62 |
| glmS | 50 | 26.90 | 6 | 19 |
| RNaseP_arch | 51 | 67.61 | 34 | 156 |
| IRES_Cripavirus | 49 | 4.92 | 7 | 36 |

Program command line parameters

In the descriptions below <data> refers to unaligned query and genome sequence in FASTA format and <query> and <genome> refer to the separate sequences also in FASTA format.

Probalign: probalign –nuc –T 7 –go 32 –ge 2 <data>

SSEARCH: ssearch –H –q –d 1 –a –f 10 –e 4 -O ssearch.out <query> <genome>

BLAST: bl2seq –p blastn –G 8 –E 6 –W 4 –S 1 –r 5 –q -4 –i <query> -j <genome>

ClustalW: clustalw –infile=<data> -outorder=input –output=fasta –outfile=cw.out

HMMER: (1) hmmbuild –nucleic –informat=PHYLIP –f –F model.hmm <query>

(2) hmmsearch model.hmm <genome>

Probalign

We first explain the maximal expected accuracy alignment methodology and how match or posterior probabilities are used. We then explain how to compute these probabilities using partition function matrices and finally tie it with expected accuracy alignment in the Probalign program.

**Posterior probabilities and maximal expected accuracy alignment**

Most alignment programs compute an optimal sum-of-pairs alignment or a maximum probability alignment using the Viterbi algorithm (Durbin *et al.,* 1998). An alternative approach is to search for the maximum expected accuracy alignment (Durbin *et al.,* 1998; Do *et al.*, 2005). The expected accuracy of an alignment is based upon the posterior probabilities of aligning residues in two sequences.

Consider sequences *x* and *y* and let *a** be their true alignment. Following the description in (Do *et al.*, 2005) the posterior probability of residue *x_i_* aligned to *y_j_* in *a** is defined as

(1)

where *A* is the set of all alignments of *x* and *y* and ***1****(expr)* is the indicator function which returns 1 if the expression *expr* evaluates to true and 0 otherwise. *P(a|x,y)* represents the probability (our belief) that alignment *a* is the true alignment *a**. From hereon we represent the posterior probability as *P*(*x_i_ ~ y_j_*) with the understanding that it represents the probability of *x_i_* aligned to *y_j_* in the true alignment *a**.

Given the posterior probability matrix *P*(*x_i_ ~ y_j_*), we can compute the maximal expected accuracy alignment using the following recursion described in Durbin *et al.*, 1998.

(2)

According to equation (1) as long as we have an ensemble of alignments *A* with their probabilities *P(a|,x,y)* we can compute the posterior probability *P*(*x_i_ ~ y_j_*) by summing up the probabilities of alignments where *x_i_* is paired with *y_j_* . One way to generate an ensemble of such alignments is to use the partition function methodology, which we now describe.

**Posterior probabilities by partition function**

Amino acid scoring matrices, normally used for sequence alignment, are represented as log-odds scoring matrices (as defined by Dayhoff *et al.*, 1978). The commonly used sum-of-pairs score of an alignment *a* (Durbin et. al., 1998) is defined as the sum of residue-residue pairs and residue-gap pairs under an affine penalty scheme.

(3)

Here *T* is a constant (depending upon the scoring matrix), *M_ij_* is the mutation probability of residue *i* changing to *j* and *f_i_* and *f_j_* are background frequencies of residues *i* and *j*. In fact, it can be shown that any scoring matrix corresponds to a log odds matrix (Karlin and Alstchul 1990; Altschul 1993).

Miyazawa 1995 proposed that the probability of alignment *a*, *P*(*a*), of sequences *x* and *y* can be defined as

(4)

where *S*(*a*) is the score of the alignment under the given scoring matrix. In this setting one can then treat the alignment score as negative energy and *T* as the thermodynamic temperature, similar to what is done in statistical mechanics. Analogous to the statistical mechanical framework, Miyazawa 1995 defined the partition function of alignments as

(5)

where *A* is the set of all alignments of *x* and *y*. With the partition function in hand, the probability of an alignment *a* can now be defined as

(6)

As *T* approaches infinity all alignments are equally probable, whereas at small values of *T*, only the nearly optimal alignments have the highest probabilities. Thus, the temperature parameter *T* can be interpreted as a measure of deviation from the optimal alignment.

The alignment partition function can be computed using recursions similar to the Needleman-Wunsch dynamic algorithm. Let *Z^M^_ij_* represent the partition function of all alignments of *x_1..i_* and *y_1..j_* ending in *x_i_* paired with *y_j_*, and *S_ij_(a)* represent the score of alignment *a* of *x_1..i_* and *y_1..j_*. According to equation (5)

(7)

where *A_ij_* is the set of all alignments of *x_1..i_* and *y_1..j_*, and *s*(*x_i_,y_j_*) is the score of aligning residue *x_i_* with *y_j_*. The summation in the bracket on the right hand side of equation (7) is precisely the partition function of all alignments of *x_1..i-1_* and *y_1..j-1_*. We can thus compute the partition function matrices using standard dynamic programming.

(8)

Here *s*(*x,y*) represents the score of aligning residue *x_i_* with *y_j_*, *g* is the gap open penalty, and *ext* is the gap extension penalty. The matrix *Z^M^_ij_* represents the partition function of all alignments ending in *x_i_* paired with *y_j_*. Similarly, *Z^E^_ij_* represents the partition function of all alignments in which *y_j_* is aligned to a gap and *Z^F^_ij_* all alignments in which *x_i_* is aligned to a gap. Boundary conditions and further details can be obtained from Miyazawa 1995.

Once the partition function is constructed, the posterior probability of *x_i_* aligned to *y_j_* can be computed as

(9)

where *Z’^M^_i,j_* is the partition function of alignments of subsequences *x_i..m_* and *y_j..n_* beginning with *x_i_* paired with *y_j_* and *m* and *n* are lengths of *x* and *y* respectively. This can be computed using standard backward recursion formulas as described in Durbin *et al.*, 1998.

In equation (9) *Z^M^_i-1,j-1_/Z* and *Z’^M^_i+1,j+1_/Z* represent the probabilities of all feasible suboptimal alignments (determined by the *T* parameter) of *x_1..i-1_* and *y_1..j-1_*, and *x_i+1.m_* and *y_j+1..n_* respectively, where *m* and *n* are lengths of *x* and *y* respectively. Thus, equation (9) weighs alignments according to their partition function probabilities and estimates *P*(*x_i_ ~ y_j_* ) as the sum of probabilities of all alignments where *x_i_* is paired with *y_j_*.

**Maximal expected accuracy alignment using partition function posterior probabilities**

Recall the maximum expected accuracy alignment formulation described earlier. In order to compute such an alignment we need an estimate of the posterior probabilities. In this report, we utilize the partition function posterior probability estimates for constructing multiple alignments. For each sequence *x, y* in the input, we compute the posterior probability matrix *P*(*x_i_ ~ y_j_*) using equation (9). These probabilities are subsequently used to compute a maximal expected multiple sequence alignment using the Probcons methodology. First, the probabilistic consistency transformation (described in detail in Do *et al.*, 2005) is applied to improve the estimate of the probabilities. Briefly, the probabilistic consistency transformation is to re-estimate the posterior probabilities based upon three-sequence alignments instead of pairwise. Note that this does not mean alignments are recomputed; our estimation (as done in Probcons) is still fundamentally based upon pairwise alignments.

After the probabilistic consistency transformation, sequence profiles are next aligned in a post-order walk along a UPGMA guide-tree. As is commonly done, UPGMA guide trees are computed using pairwise expected accuracy alignment scores. Finally, iterative refinement is performed to improve the alignment. This standard alignment procedure is described in more detail in Do *et al.,* 2005 and is implemented in the Probcons package (by the same authors).

We implement the Probalign approach by modifying the underlying Probcons program to read in arbitrary posterior probabilities for each pair of sequences in the input. All use of HMMs in the modified Probcons code is disabled. We modified the probA program of Muckstein *et al.,* 2002 for computing partition function posterior probability estimates. The Probalign program is represented algorithmically in Figure 1. Our current implementation is a beta version and mainly for proof of concept; however, the open source code is fully functional and is available with full support from http://www.cs.njit.edu/usman/probalign.

**Probalign algorithm:**

1. For each pair of sequences (*x,y*) in the input set
   1. Compute partition function matrices *Z*(*T*)
   2. Estimate posterior probability matrix *P*(*x_i_ ~ y_j_*) for (*x,y*) using equation (9)
2. Perform the probabilistic consistency transformation and compute a maximal expected accuracy multiple alignment: align sequence profiles along a guide-tree and follow by iterative refinement (Do *et. al.*).

**Fig. 1.**Probalign algorithmic description.

References

R. Durbin, S. Eddy, A. Krogh, and G. Mitchison, (1998) Biological sequence analysis: probabilistic models of proteins and nucleic acids, Cambridge University Press

C. B. Do, M. S. P. Mahabhashyam, M. Brudno, and S. Batzoglou, (2005) PROBCONS: probabilistic consistency based multiple sequence alignment. *Genome Research* 15 pp:330-340.

S. Miyazawa, (1995) A reliable sequence alignment method based upon probabilities of residue correspondences, *Protein Engineering* 8(10) pp:999-1009.

M. O. Dayhoff, R. M. Schwartz, and B. C. Orcutt, (1978) A model for evolutionary change in proteins, In M. O. Dayhoff, editor, *Atlas of Protein Sequence and Structure,* 5 pp:345-352, National Biochemical Research Foundation, Washington DC

U. Muckstein, I. L. Hofacker, and P. F. Stadler, (2002) Stochastic pairwise alignments, *Bioinformatics* 18 Suppl 2 pp:S153-160.

S. Karlin and S. F. Altschul, (1990) Methods for assessing the statistical significance of molecular sequence features by using general scoring schmes, *Proceedings of National Academy of Sciences of USA,* 87(6) pp:2264-2268

S. F. Altschul, (1993) A protein alignment scoring system sensitive at all evolutionary distances, *Journal of Molecular Evolution*, 36(3) pp:290-300
